# Optimization of Sample Construction Based on NDVI for Cultivated Land Quality Prediction

**DOI:** 10.3390/ijerph19137781

**Published:** 2022-06-24

**Authors:** Chengqiang Li, Junxiao Wang, Liang Ge, Yujie Zhou, Shenglu Zhou

**Affiliations:** 1 School of Geographic and Oceanographic Sciences, Nanjing University, Nanjing 210023, China; mg20270014@smail.nju.edu.cn (C.L.); mg1727004@smail.nju.edu.cn (L.G.); gerryzhou@smail.nju.edu.cn (Y.Z.); 2 Key Laboratory of Coastal Zone Exploitation and Protection, Ministry of Land and Resources, Nanjing 210008, China; 3 School of Public Administration, Nanjing University of Finance & Economics, Nanjing 210023, China

**Keywords:** cultivated land quality, sample construction, machine learning, NDVI, Dongtai

## Abstract

The integrated use of remote sensing technology and machine learning models to evaluate cultivated land quality (CLQ) quickly and efficiently is vital for protecting these lands. The effectiveness of machine-learning methods can be profoundly influenced by training samples. However, in the existing research, samples have mainly been constructed by random point (RPO). Little attention has been devoted to the optimization of sample construction, which may affect the accuracy of evaluation results. In this study, we present two optimization methods for sample construction of random patch (RPA) and area sequence patch (ASP). Differing from RPO samples, it aims to include cultivated land area and its size into sample construction. Based on landsat-8 Operational Land Manager images and agricultural land grading data, the proposed sample construction methods were applied to the machine learning model to predict the CLQ in Dongtai City, Jiangsu Province, China. Four machine learning models (the backpropagation neural network, decision tree, random forest (RF), and support vector machine) were compared based on RPO samples to determine the accurate evaluation model. The best machine learning model was selected to compare RPA and ASP samples with RPO samples. Results determined that the RF model generated the highest accuracy. Meanwhile, a high correlation was noted between the cultivated land area and CLQ. Thus, incorporating cultivated land area in the sample construction attributes can improve the prediction accuracy of the model. Among the three sample construction methods, the ASP yielded the highest prediction accuracy, indicating that the use of a large, cultivated land patch as the sample unit can further elevate the model performance. This study provides a new sample construction method for predicting CLQ using a machine learning model, as well as providing a reference for related research.

## 1. Introduction

As the most important means of agricultural production, cultivated land plays a critical role in human survival and development [1,2,3,4]. However, cultivated land quality (CLQ) is degrading rapidly in many regions of the world, especially in China [5]. Since economic reforms and liberalization were introduced in China, cultivated lands have been fragmented, changed to non-food croplands, and transformed to non-agricultural lands due to increasing industrialization and urbanization [6]. Furthermore, the discharge of industrial waste into cultivated lands and the excessive use of pesticides and fertilizers has also aggravated the deterioration of CLQ in China [7]. Therefore, rapid and effective methods for the evaluation of CLQ are essential to improve cultivated land resources, ensure food security, and maintain social stability [8,9,10].

Traditional methods of CLQ evaluation are based on field measurements [11], which are time-consuming and expensive. However, over the past 20 years, new CLQ evaluation techniques have been developed. In particular, remote sensing technology possesses the characteristics of wide coverage, large information acquisition, strong timeliness, rapid speed, and timely production of data to assess agricultural resources, crop growth, agricultural disasters and other information [12]. Today, remote sensing has become the main method for the rapid evaluation of CLQ, with two main avenues of study: the acquisition of relevant evaluation indices by interpreting remote sensing images [13,14] and the creation of an inversion model using remote sensing images [15,16]. The evaluation of CLQ is also conducted extensively using machine learning techniques [17,18]. This data mining approach mainly involves the establishment of relationships between the CLQ and evaluation factors based on training samples. In this approach, the subjectivity of assigning weights to evaluation indices is eliminated [19], substantially improving the efficiency of the CLQ evaluation. Many machine learning models are available, but those most frequently used for CLQ evaluation include backpropagation neural network (BPNN) [20,21], decision tree (DT) [22,23], random forest (RF) [18,24], and support vector machine (SVM) [25,26].

In recent years, remote sensing technology and machine learning models have been combined to rapidly and efficiently evaluate CLQ. Li et al. (2020) extracted spectral information on cultivated land from Landsat-Thematic Mapper (TM) images and used the extreme learning machine model to evaluate CLQ in hilly areas of central-south Shandong Province, China [27]. Liu et al. (2019) extracted and screened the vegetation index using GF-1 remote sensing imagery to produce a cultivated land fertility index and used the BPNN model to evaluate the CLQ in the Conghua District of Guangzhou City, Guangdong Province, China [28]. Even though these studies have demonstrated the process of rapid evaluation of CLQ, they use only one sample construction method for evaluation. The evaluation accuracy of a machine learning method depends on the training samples [29,30]. Thus, the quality of the sample data profoundly influences the learning ability of these models. The construction of samples has predominantly been based on spatial points [15,28,31], while cultivated land area has been neglected, despite previous studies demonstrating that CLQ is correlated with cultivated land area [32,33]. Therefore, studies aimed at optimizing the construction of samples for the evaluation of CLQ using machine learning models are required.

The objectives of this study were as follows: (1) to select the machine learning model with the best prediction potential based on the random point (RPO) sample construction method; (2) to optimize the sample construction approach and determine whether the prediction accuracy of a model can be improved by incorporating the cultivated land area; and (3) to determine the sample construction method with the highest prediction accuracy. The results of this study provide a guide for the rapid and efficient prediction of regional CLQ and for the enhancement of cultivated land protection.

## 2. Materials and Methods

### 2.1. Study Area

**Figure 1:**
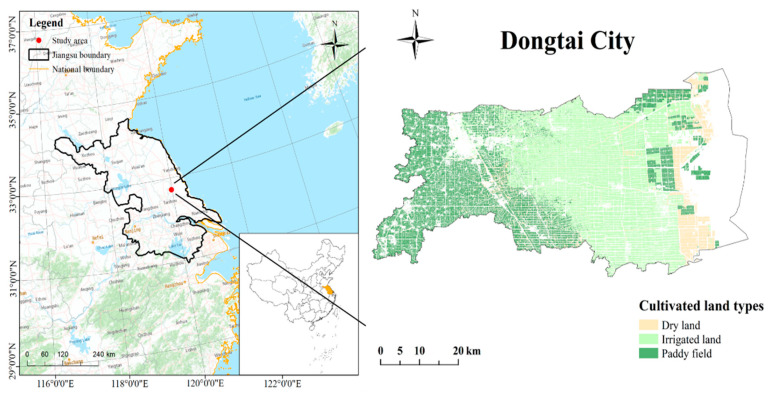
Map showing the location of the study area.

Dongtai City is in the center of the coastal area in Jiangsu Province, China (Figure 1), extending from longitudes 120°07′ to 120°53′ E and latitudes 32°33′ to 32°57′ N. The city has a subtropical monsoon maritime climate, with an average annual temperature of 14.6 °C and an average annual precipitation of 1061.2 mm. As a typical agricultural city in the eastern coastal plain of China, Dongtai is mostly flat and rich in cultivated lands. In 2020, cultivated lands occupied approximately 136,900 ha, accounting for nearly 43.1% of the total land area covered by the city. Therefore, the protection of CLQ has been considered in policies during the development of the city. To formulate an adequate protection plan, fast and efficient methods for the evaluation of CLQ are required.

### 2.2. Data Collection and Processing

The CLQ data of field verification points were obtained from the 2018 CLQ grading achievement database of Dongtai City, provided by the Bureau of Natural Resources and Planning. This achievement was based on using the cultivated land patch of Dongtai City 2018 1:5000 land use status map as the evaluation unit, and the evaluation method and parameter system adopted were determined according to the cultivated land quality evaluation regulation “Agricultural Land Quality Grading Regulation” (GB/T 28407-2012) issued by the Ministry of Land and Resources of China. Nine evaluation factors were considered: soil organic matter, PH value, soil salinization degree, irrigation guarantee rate, drainage condition, barrier layer depth from the surface, soil erosion degree, surface soil texture, and effective soil layer thickness. The data for the nine factors were obtained through laboratory measurements or field surveys. This result is a relatively complete background data of CLQ in Dongtai City, and the CLQ in Dongtai City is divided into levels 1–4. 

The normalized difference vegetation index (NDVI) accurately highlights the extent of vegetation cover and the fertility of cultivated lands in a given region [16,34]. For the prediction of CLQ in the study, we used 2018 Landsat 8 Collection 1 Tier 1 8-day NDVI Composite data with a resolution of 30 m; the data were obtained from the United States Geological Survey (USGS; https://www.usgs.gov/land-resources/nli/landsat/landsat-data-access, accessed on 10 March 2021).

**Figure 2:**
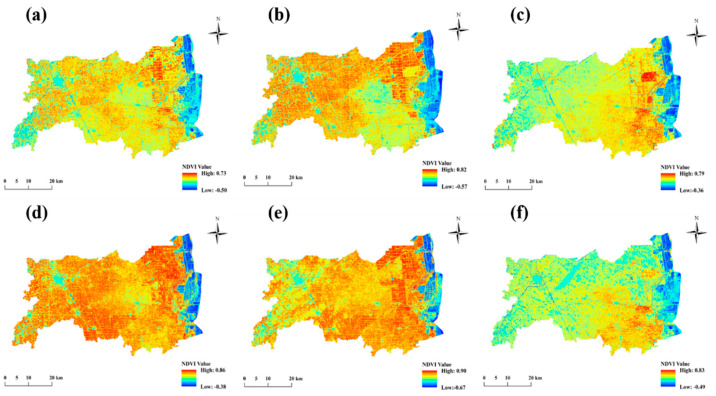
Diagrams showing the 2−month average NDVI images for the study area in 2018, including (**a**) January to February, (**b**) March to April, (**c**) May to June, (**d**) July to August, (**e**) September to October, and (**f**) November to December.

To improve the accuracy of the CLQ level prediction, the annual image was divided into 12 months, and 12 images were synthesized. The study area has a high level of cloud cover, and so the NDVI values were split into groups representing 2 months, generating six images (Figure 2) to minimize the effect of cloudy conditions and ensure accuracy. The cultivated land type and cultivated patch area were also selected as independent variables for the models. These data were obtained from the 2018 CLQ grading achievement database for Dongtai City. In the present study, the mean NDVI values for January to February, March to April, May to June, July to August, September to October, and November to December, as well as the type and area of cultivated land were independent variables for the models [9,33,35,36].

### 2.3. Machine Learning Models

CLQ levels in the study area were simulated and predicted using four machine learning models (BPNN, DT, RF, and SVM) that are commonly employed in CLQ evaluation. These models were constructed using the model function construction package in the SPSS Modeler 18.0 (IBM) software. The following is a brief introduction to the four models.

(1) Backpropagation neural network model

The BPNN is a general supervised machine learning model, which consists of three layers: the input layer, hidden layer, and the output layer [37]. It learns by signal forward propagation and error backpropagation, adjusting the weights in each successive layer to reduce the errors at each level, and finally outputs prediction or classification results [38]. In this study, a BPNN model was constructed by using the BPNN construction function package in SPSS Modeler 18.0 software (Chicago, IL, USA).

(2) Decision Tree model

The DT is a common classification and regression algorithm, and the operation process involves dividing into the root, intermediate, and leaf nodes [39]. The root node is the sum of all datasets predicted by the model, while the intermediate node represents the division of the selected dataset based on defined rules, and the leaf node is the output of the result of the model. In this study, a DT model was constructed by the DT construction function package in SPSS Modeler 18.0 software.

(3) Random Forest model

The RF model is an ensemble-learning algorithm proposed by Breiman [40]. The principle involves the gathering of decision trees via “bagging”, generating prediction results, and building a prediction model based on the binary splitting of prediction variables [41]. This model can then be used for classification, clustering, and regression. In the RF model, the bootstrap sampling method is used for the selection of samples, and a DT model based on data from each sample is then constructed. The prediction results of multiple DT models are then combined, and the prediction result is obtained through the voting evaluation. In this study, an RF model was constructed by employing the RF construction function package in SPSS Modeler 18.0 software.

(4) Support Vector Machine model

The SVM is a classical nonparametric machine learning model, which was originally limited to the binary classification problem [42]. Based on a hyperplane, the unclassified dataset is divided into discrete categories that are consistent with the training set, such that the distance between blank areas of the two categories is maximized when the accuracy is maximized. At present, the kernel function associated with the SVM model has been extended to accommodate multiple classifications. In this study, an SVM model was constructed by using the SVM construction function package in SPSS Modeler 18.0 software, with the radial basis function selected as the kernel function.

### 2.4. Sample Construction

To improve the accuracy of CLQ prediction using machine learning models, we optimized the sample construction method. Sample construction was performed using the random patch (RPA) and area sequence patch (ASP) methods, and these were compared with the construction based on the commonly used random point (RPO) method. The sampling methods are described briefly further.

(1) Random point sampling

**Figure 3:**
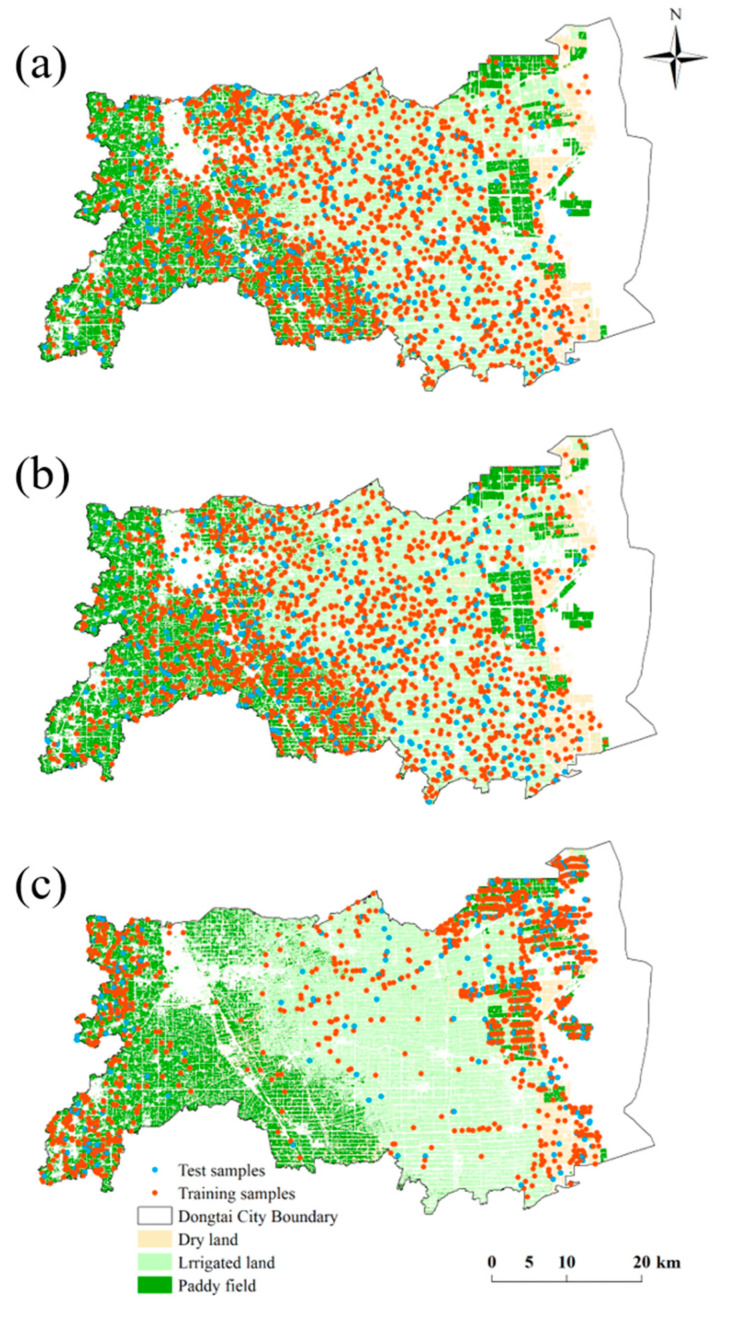
Map showing the distribution of three samples. (**a**) RPO samples, (**b**) RPA samples, (**c**) ASP samples.

RPO sampling is the most common sample construction method used for CLQ evaluation. In the CLQ level map of Dongtai City, 2000 points were randomly generated to produce the RPO samples (Figure 3a). Each point contains information on cultivated land type and the average 2-month NDVI value of the cultivated land.

(2) Random patch sampling

Cultivated land is actually a polygon, not a point. It has area, which is highly correlated with CLQ [32,33,36]. Therefore, considering a cultivated land patch as the unit, in the CLQ level map of Dongtai City, 2000 cultivated land patches were randomly selected to generate the RPA samples (Figure 3b). Each patch contains information on cultivated land type, cultivated land area, and the 2-month average NDVI value of cultivated land.

(3) Area sequence patch sampling

In general, agricultural production tends to take place on large and concentrated cultivated land. It is important to further highlight the influence of the cultivated land area on the CLQ. In the CLQ level map of Dongtai City, a large, cultivated land patch was considered as the unit. A total of 2000 cultivated land patches were selected according to the area sequence from large to small to create the ASP samples (Figure 3c). As with the RPA samples, each patch contains the information of cultivated land type, cultivated land area, and the 2-month average NDVI value of the cultivated land.

### 2.5. Model Validation and Evaluation

#### 2.5.1. Training Set and Test Set

The algorithm flow was established according to the characteristics of the SPSS Modeler 18.0 software, which included an input node, type node, partition node and model node. After the entire input dataset was classified, the whole dataset was divided according to random seeds by partition nodes. In this study, 80% of each sample were training sets for establishing evaluation models and 20% were test sets used to validate the model [43,44]. To ensure the repeatability of model prediction, the data were randomly classified according to random seeds.

#### 2.5.2. Model Evaluation Index

A confusion matrix is often used to visualize the performance of a classification model. Table 1 summarizes the content of a confusion matrix, and a true positive (TP) indicates that the machine learning model predicts a positive class and that the actual class is a positive class too. A false negative (FN) shows that the machine learning model predicts a negative class, but the actual class is positive. A false positive (FP) shows that the machine learning model predicts a positive class, but the actual class is negative. Finally, a true negative (TN) demonstrates that the machine learning model predicts a negative class, and the actual class is negative too. Based on these four parameters, the following performance evaluation indicators of a model can be obtained:

Accuracy: This metric represents the percentage of samples that predict correctly.

(1) $$\mathrm{Accuracy}=\frac{\mathrm{TP}+\mathrm{TN}}{\mathrm{TP}+\mathrm{FP}+\mathrm{FN}+\mathrm{TN}}$$

Precision: This indicator denotes the proportion of samples correctly predicted for a given category to the total number of samples predicted for that category.

(2) $$\mathrm{Precision}=\frac{\mathrm{TP}}{\mathrm{TP}+\mathrm{FP}}$$

Recall: This indicator represents the proportion of samples that are correctly predicted for a given category.

(3) $$\mathrm{Recall}=\frac{\mathrm{TP}}{\mathrm{TP}+\mathrm{FN}}$$

F1-score: This metric is the harmonic mean of the recall and precision and helps to determine the accuracy and robustness of a classification model.

(4) $$F1-\mathrm{score}=\frac{2*\mathrm{Recall}*\mathrm{Precision}}{\mathrm{Recall}+\mathrm{Precision}}$$

Based on the test set, the accuracy of machine learning model prediction results was evaluated by using the 2018 CLQ grading achievement database of Dongtai City. The classification performance of the model was evaluated by comparing the accuracy, precision, recall, and F1-score of the model. The value range of accuracy, precision, recall, and F1-score were 0–1. The higher the accuracy, precision, recall, and F1-score, the better the classification effect and prediction ability of the model [43]. In the prediction of multi-classification problems, the precision, recall, and F1-score are calculated separately for each category, and the averages are then denoted as the macro-precision, macro-recall and macro-F1 score.

### 2.6. Establishment of Research Program

**Figure 4:**
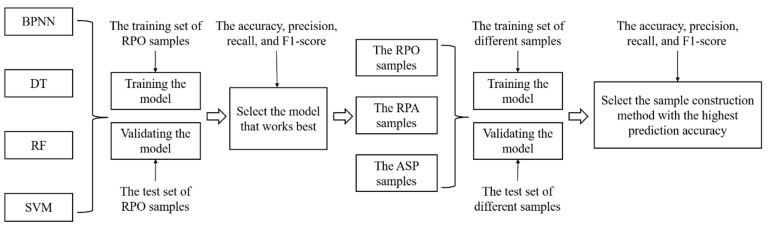
Map showing the establishment of research program.

In this study, we present two optimization methods for sample construction of RPA and ASP and compared with commonly used RPO samples. In addition, different machine learning models were compared to improve the prediction accuracy. The overview framework of this study is shown in Figure 4.

The specific steps were as follows:

(1) Four machine learning models (BPNN, DT, RF, SVM) commonly used in cultivated land quality evaluation were selected.
(2) The training set of RPO samples was used to train the model. Training the model was stopped when the simulation accuracy of the model training set was no longer improved, and the optimal model was formed. The test set of RPO samples was used to verify the model.
(3) The accuracy, precision, recall and F1-score of different models were calculated, and the model with the best classification effect was selected.
(4) RPA samples and ASP samples were applied to the machine learning model with the best performance and compared with RPO samples.
(5) The model was trained with the training set of RPA samples and ASP samples, respectively. Then, the model was validated using the test sets of RPA samples and ASP samples, respectively.
(6) The accuracy, precision, recall and F1-score of the model under different sample construction methods were calculated, and the sample construction method with the highest prediction accuracy was selected.

## 3. Results

### 3.1. Model Screening Based on the RPO Samples

Following preprocessing of the RPO sample dataset in the SPSS Modeler 18.0 software, the machine learning models BPNN, RF, DT, and SVM, were used to simulate and predict the CLQ levels in the study area. The overall accuracy of data associated with the models is presented in Table 2. Regarding the training dataset, the highest accuracy (79.5%) was obtained from the RF model, while the lowest (64.4%) was linked to the DT model. For the test dataset, the SVM model showed the highest accuracy (63.0%), while the RF model produced the lowest accuracy (57.7%). The RF model displayed the highest difference in accuracy between the training and test datasets, indicating overfitting.

**Figure 5:**
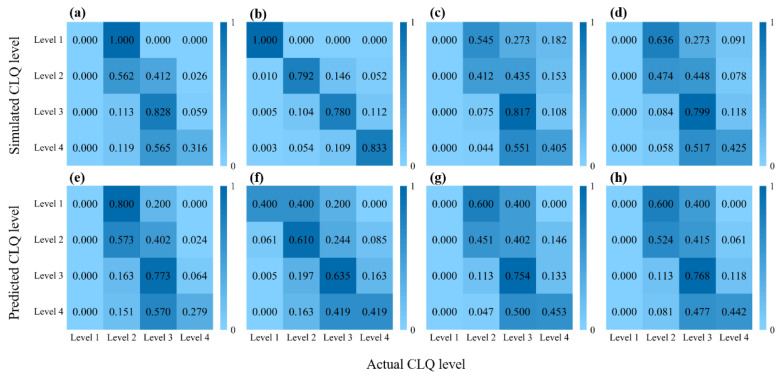
Results from four machine learning models showing the confusion matrix of the (**a**) BPNN training dataset, (**b**) RF training dataset, (**c**) DT training dataset, (**d**) SVM training dataset, (**e**) BPNN test dataset, (**f**) RF test dataset, (**g**) DT test dataset, and (**h**) SVM test dataset.

The simulation and prediction results associated with the four models for different levels of cultivated land were generated, and the corresponding confusion matrices were created. According to the data shown in Figure 5, excluding the RF model, the other models inadequately extracted the data required for classifying Level 1 cultivated land during training. Therefore, an accurate prediction of Level 1 cultivated land was impossible using these models, with both the training and test datasets producing accuracy values of 0. Regarding Level 2 cultivated land, the accuracy values of the training and test datasets for the RF model were the highest (79.2% and 61.0%, respectively), while those of the DT model were the lowest (41.2% and 45.1%, respectively). For Level 3 cultivated land, all four models produced high accuracy values for both the training and test datasets. The BPNN model produced the highest accuracy values for the training and test datasets (82.8% and 77.3%), while the RF model yielded the lowest (78.0% and 63.5%, respectively). In contrast, for Level 4 cultivated land, all four models generated low accuracy values.

**Figure 6:**
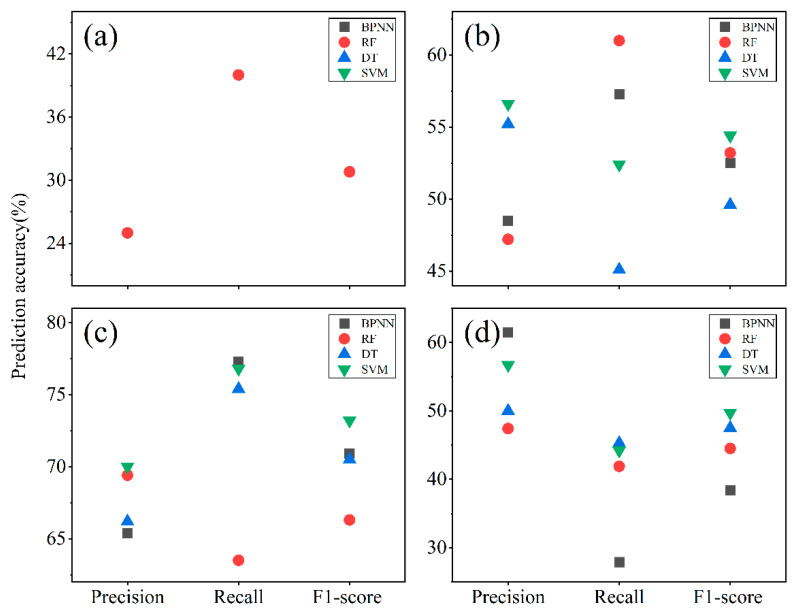
Plot showing comparisons of the prediction accuracy values for four machine learning models associated with different levels of cultivated land including (**a**) Level 1 (the BPNN, DT, and SVM models involves loss of some data, and the missing precision, recall, and F1-score are assigned values of 0), (**b**) Level 2, (**c**) Level 3, and (**d**) Level 4.

Figure 6 displays the prediction accuracies of the four models when compared based on the precision, recall, and F1-score of the predicted results. Based on the F1-score for all cultivated land levels, the macro-F1 scores for the four models follow the order RF (48.7%) > SVM (44.3%) > DT (41.9%) > BPNN (40.5%), with the highest prediction ability being attributed to the RF model. These are consistent with the results from the macro-precision and macro-recall.

According to the performance evaluation indices of the machine learning models, the SVM model displayed the highest prediction accuracy, while the RF model had the highest prediction F1-score. Although the RF model exhibits overfitting, the model can learn the data of cultivated land levels for a small number of samples. Therefore, loss of cultivated land level data was prevented; as such, the applicability of the RF model is better than that of the other three models.

### 3.2. Optimization of the Sample Construction 

In Section 3.1, the RF model was shown to produce the best prediction. However, the prediction accuracy associated with the RPO samples was low, and optimization of the sample construction was required. Herein, the cultivated land area was added to the sample attributes to generate RPA and ASP samples. The RF model was then used to simulate and predict CLQ levels; the accuracy values associated with the two-sample datasets are presented in Table 3. The accuracy of the training set for the RPA-RF model was 79.0%, whereas that for the test set was 63.5%. The significant difference between these accuracy values indicates that the model involves overfitting. In contrast, the training and test datasets for the ASP-RF model produced values of 90.1% and 86.1%, respectively. The small difference between the accuracy values indicates that the model is associated with good fitting.

**Figure 7:**
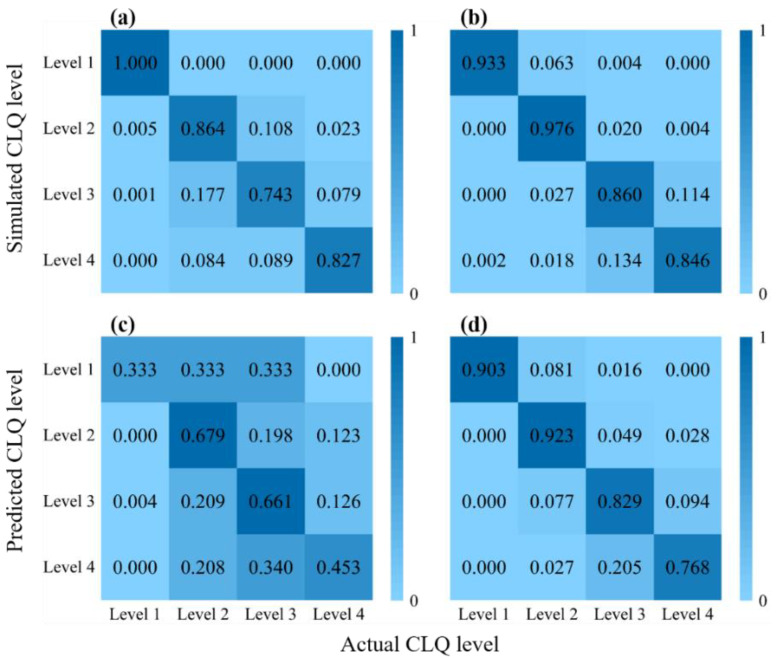
Confusion matrices of results for the (**a**) simulation using the RPA-RF model, (**b**) simulation using the ASP-RF model, (**c**) prediction using the RPA-RF model, and (**d**) prediction using the ASP-RF model.

The simulation and prediction results of the RPA-RF and ASP-RF models were obtained, and the corresponding confusion matrices were created. Figure 7 shows that the RPA-RF model adequately simulated all levels of cultivated land, and the associated accuracy values for the training datasets exceeded 70%. However, the prediction performance of the RPA-RF model varied significantly for different levels of cultivated land. The accuracy values from the test datasets for Level 1 and Level 4 cultivated lands were 33.3% and 45.3%, while those for Level 2 and Level 3 were 67.9% and 66.1%, respectively. The RPA-RF model was characterized by overfitting during learning of levels 1, 2, and 4 cultivated lands. In contrast, the ASP-RF model produced adequate simulations and predictions for all levels of cultivated land. The accuracy values of the simulation and prediction for different levels of cultivated land were high, and the fitting effect was good.

The performances of the RPA-RF and ASP-RF models were further evaluated based on the precision, recall, and F1-score of the predicted results (Table 4). Using the F1-score as an example, the RPA-RF model produced the highest prediction accuracy (72.0%) for Level 3 cultivated land and the lowest prediction accuracy (40.0%) for Level 1 cultivated land, with the prediction accuracy of the macro-F1 score being 53.2%. In contrast, the ASP-RF model produced high F1-score for all levels of cultivated land, and the prediction accuracy of the macro-F1 score was 85.9%.

### 3.3. Optimal Sample Construction 

**Figure 8:**
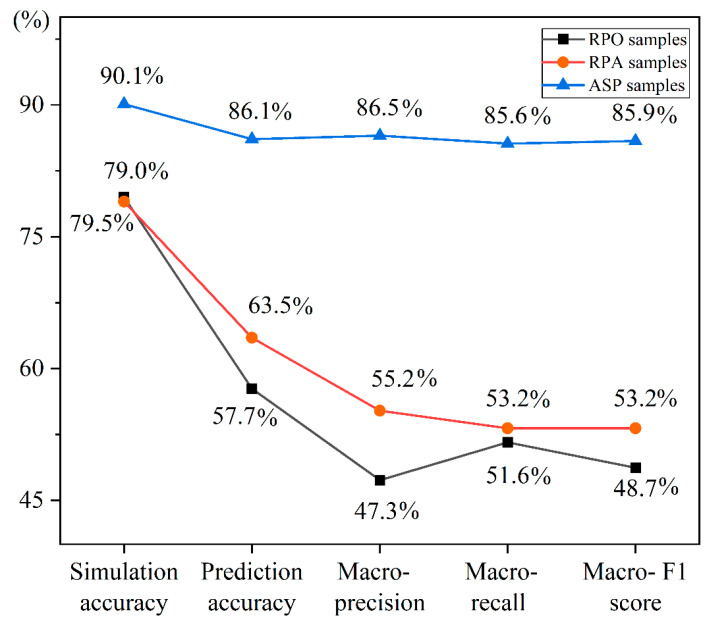
Comparison of the performance of the RPO-RF, RPA-RF, and ASP-RF models.

The performance evaluation indices of the RPO-RF, RPA-RF, and ASP-RF models were compared to highlight the best sample construction method for predicting CLQ level. Figure 8 shows that the prediction results of the RPO-RF and RPA-RF models were similar. However, compared with the RPO-RF model, the prediction accuracy, macro-precision, macro-recall, and macro-F1 score of the prediction results for the RPA-RF model increased by 5.8%, 7.9%, 1.6%, and 4.5%, respectively. These values indicate that the prediction results associated with the RPA samples are more accurate than those obtained using the RPO samples, and the performance of the model was improved. The ASP-RF model performed better for all indicators. Compared with the RPO-RF model, the prediction accuracy, macro-precision, macro-recall, and macro-F1 score of the model increased by 28.4%, 39.2%, 34.0%, and 37.2%, respectively. These values demonstrate that ASP sampling further improves the performance of the model and is the best sample construction method for the prediction of the CLQ level.

## 4. Discussion

### 4.1. Selection of CLQ Evaluation Methods

The rapid and efficient evaluation of CLQ is currently considered a requirement for cultivated land resource management [45]. Several studies on CLQ evaluations based on NDVI have produced good results. Guan et al. used NDVI data extracted from Landsat 8 multispectral images to produce a CLQ inversion model, with an accuracy of 93.6% [16]. Previous studies have demonstrated the feasibility of predicting the CLQ level based on the NDVI. However, most studies only select remote sensing images of a single month to obtain NDVI [28], which may have accidental factors and affect the accuracy of the CLQ evaluation. In the present study, images of the study area for a whole year were used to generate NDVI data according to the month in order to improve the accuracy of the evaluation results.

Machine learning techniques can substantially enhance CLQ evaluation efficiency, but the prediction effect will be different with different models. In this study, the BPNN, DT, RF, and SVM models were compared for the prediction of CLQ levels. The RF model produced the highest prediction accuracy and the best classification. This finding is consistent with many similar studies. Zhang et al. compared the performances of the multinomial logistic regression, k-nearest neighbor (KNN), and RF models in the prediction of soil category and reported that the RF model produced superior results [30]. Ge et al. evaluated the accuracy of different machine learning models for the classification of land cover in arid regions of China and noted that the RF model generated more accurate classification compared with the KNN, SVM, and artificial neural network models [46]. Studies using other machine learning models to predict CLQ have also achieved satisfactory results [47]. The reason could be related to the type of variable used in the study. In general, the performance of a machine learning model depends on the sample data. Certain machine learning models perform better than others, owing to the relationships between variables and outputs [43]. Linear models such as SVM provide better results if there is a linear relationship between the variables and the output [48]. For complex and nonlinear relationships, DT-based models (e.g., RF) may perform better than linear models [49]. In the current study, RF performed better than SVM, indicating that the relationships between the different variables and CLQ levels were nonlinear and complex.

RF performs better than other nonlinear models (e.g., DT and BPNN) when dealing with complex nonlinear relations. The reasons for this performance difference are as follows. RF models improve consistency by aggregating multiple models to minimize the instability of a single tree model [50]. In contrast, the DT model only uses a single tree to learn complex relationships among CLQ levels and variables. A low prediction accuracy implies that the DT model cannot handle such complex relationships. The DT model is very unstable, and small changes in the learning sample can produce completely different trees [44]. The BPNN model is based on a gradient descent algorithm, which randomly initializes the connection weights and thresholds of each layer into 0–1 values before starting training. In the face of complex nonlinear relationships, such unoptimized random initial values tend to slow the convergence speed of the BPNN model and make the final result easily non-optimal [51]. Furthermore, our results also show that the BPNN, DT, and SVM models are characterized by information loss during the simulation and prediction of Level 1 cultivated land. This limitation may be attributed to the low proportion of Level 1 cultivated land in the RPO samples. If the training data set is small, the model cannot learn the general principles, and so the performance will be unsatisfactory [52]. However, due to the high data use rate, the RF model is suitable for mining the information required for simulation and prediction using limited samples [44]. Therefore, the applicability of this model is better than that of the other three.

### 4.2. Effect of the Sample Construction Method on the Model Prediction Accuracy

The quality of the sample dataset profoundly affects the performance of machine learning models. Therefore, optimizing the sample construction method is an effective way to improve the prediction accuracy of these models [53]. Herein, the cultivated land area was included in the sample attributes, and a cultivated land patch was the sample unit during the construction of the RPA samples. The macro-precision, macro-recall and macro-F1 score of the RPA-RF model were higher than those of RPO-RF model. This result indicates a high correlation between the cultivated land area and CLQ level and that including the cultivated land area into the sample attribute can improve the performance of the model. Zeng et al. analyzed the degree of correlation between the CLQ and its influencing factors in Xiangyang City, Hubei Province, China, by using the grey correlation degree analysis method and found that the CLQ was significantly correlated with the cultivated land area, with a correlation degree of 0.74 [36]. However, Lin et al. analyzed the influencing factors of CLQ in Wulan County, Qinghai Province, China, and found no significant correlation between cultivated land area and CLQ [54]. This discrepancy may be caused by the difference in natural and social environments between the two regions. The CLQ in the eastern Plain of China may be more sensitive to area factors than that in the western plateau of China. 

To further evaluate the influence of the cultivated land area on the CLQ, a large, cultivated land patch was used as the sample unit to construct the ASP samples. The macro-precision, macro-recall, and macro-F1 score of the ASP-RF model were higher than those of the RPA-RF and RPO-RF models. This result indicates that model performance can be further improved by building samples with large, cultivated land patches as units. Sheng et al. evaluated the quality of cultivated land on the alluvial fan in Jimusar County, Xinjiang, China, based on different cultivated land evaluation units and found that when large areas of cultivated land were used as the evaluation units for CLQ, the evaluation result was accurate [31]. The reason may be that a large area of cultivated land is more representative of the characteristics of CLQ. This representativeness enhances the training of the model and improves performance.

The differences in macro-precision, macro-recall, and macro-F1 score of the four models were small. Taking the macro-F1 score as an example, the values of the four models ranged from 40.5% to 48.7%. This finding is consistent with that of many similar studies. Chagas et al. predicted soil types on the tropical slopes of Brazil: the overall accuracy of the RF model was 78.8%, while the overall accuracy of the DT model was 70.2% [55]. Du et al. predicted the soil type of Heshan Farm in Heilongjiang Province, China, and found that the overall accuracy of the DT model was 56.4%, that of the logistic regression model was 50.4%, and that of the SVM model was 50.5% [29]. However, the macro-F1 score differences between the three sample construction methods were large, ranging from 48.7% to 85.7%, suggesting that the quality of the sample dataset is more important than the model itself [55,56].

### 4.3. Implications for Policy and Decision Making

China’s state policy is to protect food security and safeguard the red line of 1.8 billion mu cultivated land, and the quality of cultivated land plays a fundamental role [57]. Cultivated land resources possess characteristics of wide distribution and large area, and the quantity and quality of cultivated land greatly changes with time [28]. Therefore, to strengthen the control and construction of cultivated land, it is necessary to timely and accurately understand the quality and spatial distribution of cultivated land. In this study, the proposed ASP sample construction method further improved the accuracy of predicting CLQ using machine learning. A rapid and efficient CLQ evaluation method can provide a timely and accurate basis for orderly demarcation of permanent basic farmland, occupation or protection compensation of cultivated land, and calculation of compensation for expropriation of land [31]. Furthermore, land use planning and land reclamation planning can manage cultivated land differently, according to the spatial distribution of CLQ grade [58]. In addition, farmers and farms can timely and effectively adjust agricultural input according to the quality of cultivated land, improving agricultural production efficiency.

### 4.4. Research Limitations and Prospects

In the present study, a sample construction method for evaluating CLQ using a machine learning model was optimized. The proposed ASP sample construction method can improve the prediction accuracy of machine learning models. In our future research, examining how to determine the cultivated land area threshold in sample construction, instead of the area sequence, will be an important research issue. It has to be pointed out that to quickly and efficiently evaluate the quality of cultivated land, this study selected fewer variables. In the future, we will introduce other variables that are closely linked to the CLQ, to further optimize the sample construction method to achieve a prediction that is more accurate.

## 5. Conclusions

The combination of remote sensing technology and machine learning models enables rapid and effective prediction of CLQ levels, while the optimization of the sample construction can enhance the accuracy of the prediction results. In this study, the prediction accuracy of three sample generation methods was compared using machine learning model. The conclusions are as follows: (1) Based on the RPO sampling method, the RF model produced the highest overall accuracy relative to the BPNN, DT, and SVM models, and exhibited the best application effect. (2) The prediction accuracy of the RPA-RF model surpassed that of the RPO-RF model, which indicated that inclusion of the cultivated land area into the sample construction attributes improved the prediction potential of the model. (3) Among the three sample types, the ASP-RF model yielded the highest prediction accuracy, which suggests that the use of a larger cultivated land patch as the sample unit can further enhance the prediction of the model. This study provided a new sample construction method for evaluating CLQ using a machine learning model, as well as providing a reference for related research.

## Figures and Tables

**Table 1:** Confusion matrix for the binary classification.

		Actual	
		Positive	Negative
Predicted	Positive	True Positive (TP)	False Positive (FP)
	Negative	False Negative (FN)	True Negative (TN)

**Table 2:** Accuracy data for the training and test sets for four models.

Machine Learning Model	The Accuracy of Training Dataset	The Accuracy of Test Dataset
BPNN	66.4%	60.6%
RF	79.5%	57.7%
DT	64.4%	60.9%
SVM	65.0%	63.0%

**Table 3:** Accuracy data for the training and test datasets of the RPA-RF and ASP-RF models.

Samples	The Accuracy of Training Dataset	The Accuracy of Test Dataset
RPA-RF	79.0%	63.5%
ASP-RF	90.1%	86.1%

**Table 4:** Prediction accuracy data for the RPA-RF and ASP-RF models.

CLQ Level	Sample Construction Method	Precision	Recall	F1-Score
Level 1	RPA-RF	50.5%	33.3%	40.0%
	ASP-RF	100%	90.3%	94.9%
Level 2	RPA-RF	54.5%	67.9%	60.5%
	ASP-RF	85.6%	92.2%	88.8%
Level 3	RPA-RF	79.2%	66.1%	72.0%
	ASP-RF	75.2%	82.9%	78.9%
Level 4	RPA-RF	36.4%	45.3%	40.3%
	ASP-RF	85.1%	76.8%	80.8%
Macro Average	RPA-RF	55.2%	53.2%	53.2%
	ASP-RF	86.5%	85.6%	85.9%

## Data Availability

Not applicable.
